# Highly efficient light harvesting of a Eu(iii) complex in a host–guest film by triplet sensitization†

**DOI:** 10.1039/d3sc01817b

**Published:** 2023-05-29

**Authors:** Shiori Miyazaki, Kenichi Goushi, Yuichi Kitagawa, Yasuchika Hasegawa, Chihaya Adachi, Kiyoshi Miyata, Ken Onda

**Affiliations:** a Department of Chemistry, Kyushu University 744 Motooka, Nishi Fukuoka 819-0395 Japan kmiyata@chem.kyushu-univ.jp konda@chem.kyushu-univ.jp; b Center for Organic Photonics and Electronics Research (OPERA), Kyushu University 744 Motooka, Nishi Fukuoka 819-0395 Japan; c International Institute for Carbon Neutral Energy Research (I2CNER), Kyushu University 744 Motooka, Nishi Fukuoka 819-0395 Japan; d Faculty of Engineering, Hokkaido University N13W8, Kita-ku Sapporo Hokkaido 060-8628 Japan; e Institute for Chemical Reaction Design and Discovery (WPI-ICReDD), Hokkaido University N21W10, Kita-ku Sapporo Hokkaido 001-0021 Japan

## Abstract

Trivalent lanthanide complexes are attractive light emitters owing to their ideal high color purity. Sensitization using ligands with high absorption efficiency is a powerful approach to enhancing photoluminescence intensity. However, the development of antenna ligands that can be used for sensitization is limited due to difficulties in controlling the coordination structures of lanthanides. When compared to conventional luminescent Eu(iii) complexes, a system composed of triazine-based host molecules and Eu(hfa)_3_(TPPO)_2_ (hfa: hexafluoroacetylacetonato and TPPO: triphenylphosphine oxide) significantly increased total photoluminescence intensity. Energy transfer from the host molecules to the Eu(iii) ion occurs *via* triplet states over several molecules, according to time-resolved spectroscopic studies, with nearly 100% efficiency. Our discovery paves the way for efficient light harvesting of Eu(iii) complexes with simple fabrication using a solution process.

## Abstract 2

Very high photoluminescence intensity of a Eu(iii) complex is achieved using a host–guest system. The intensity is 400 times larger than that of the Eu(iii) complex itself.

## Graphical abstract

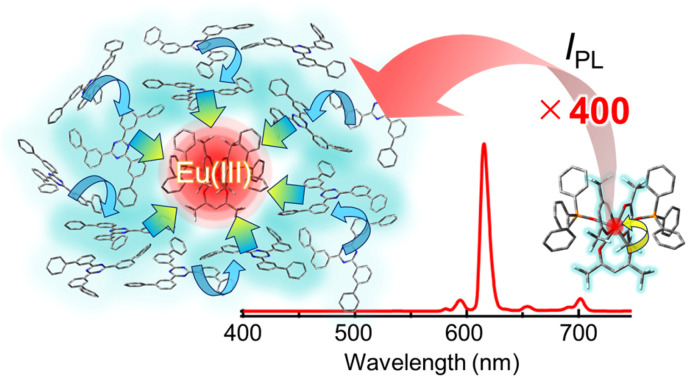

## Introduction

Molecule-based light-emission technologies have been intensively developed over the last few decades owing to their various applications, such as display panels, bioimaging sensors, optical telecommunications, and laser diodes.^1–3^ Light-emitting materials must emit narrow-band light to achieve high color purity in their applications. However, general organic molecular emitters exhibit broadband emissions with a full width at half-maximum (FWHM) of 70–100 nm. Trivalent lanthanide (Ln(iii)) complexes exhibit narrow-band emissions with FWHMs of 10–20 nm caused by transitions between f-orbitals in Ln(iii) that are shielded by electrons in the occupied 5s and 5p orbitals;^4^ however, direct photoexcitation of the Laporte-forbidden f–f transition in Ln(iii) with a small absorption coefficient (*ε* < 10 M^−1^ cm^−1^) is difficult.^5^ Many efforts have been made to overcome this difficulty by synthesizing organic ligands with large absorption coefficients and appropriate energy levels, which play an important role in efficient photosensitizers and intra-molecular ligand-to-Ln(iii) energy transfer in complexes.^6–8^

The overall photoluminescence (PL) intensity (*I*_PL_)^9,10^ of an Ln(iii) complex in a dilute solution or thin film is expressed as

1 *I*_PL_ = *ε*_ligands_ × *ϕ*_tot_

where *ε*_ligands_ and *ϕ*_tot_ represent the sum of all ligand absorption coefficients and the overall luminescence quantum yield of Ln(iii) from ligand photoexcitation *via* intra-molecular energy transfer, respectively. This equation indicates that high PL intensity requires high light-absorption ability and luminescence quantum yield. Eu(iii)(hfa)_3_(DPPTO)_2_ (hfa: hexafluoroacetylacetonato and DPPTO: 2-diphenyl phosphoryl triphenylene Fig. S1†) is one of the Ln(iii) complexes with the highest luminescence intensity in solution, *I*_PL_ = 90 000 M^−1^ cm^−1^. The following three factors contribute to this high *I*_PL_: (i) high *ε*_ligands_ = 170 000 M^−1^ cm^−1^ of the two DPPTO ligands owing to their triphenylene chromophores,^11^ (ii) high *ϕ*_tot_ = 0.53 owing to suppression of nonradiative decay due to low vibrational frequencies of the phosphine-oxide linker in the DPPTO ligand and CF bonds in the hfa ligand,^12–16^ and (iii) enhancement of transition intensities in Eu(iii) due to the asymmetric structure^17–21^ formed by the hfa and DPPTO ligands. However, further improvement of luminescence intensity by designing new ligands is limited due to the difficulty of synthesizing ligands that simultaneously contain multiple chromophores with a large absorption coefficient and stable coordination to Ln(iii) compared to general transition metal ions.

We propose that a host–guest system, composed of π-conjugated molecules and Ln(iii) complex emitters, is an ideal system for drastically increasing the PL intensity of Ln(iii) complexes while requiring minimal fabrication. Multiple π-conjugated molecules with high absorption coefficients serve as antennae for photosensitizing Ln(iii) complexes in this system; therefore, a much higher absorption coefficient, denoted by *ε*_hosts_ in this case, is expected in eqn (1) when compared to the molecular Ln(iii) complex. Moreover, no linkers were required to coordinate Ln(iii). However, to achieve high *ϕ*_tot_, such a host–guest system must overcome additional challenges; not only intra-molecular energy transfer but also inter-molecular energy transfer processes from host molecules to the Ln(iii) complex are involved, and each process must be highly efficient. To achieve a very high *I*_PL_ for the host–gust system, it is essential to understand the mechanisms of the entire energy transfer process and design lossless energy transfer processes. To address these concerns, we chose a simple Eu(iii) complex, Eu(iii)(hfa)_3_(TPPO)_2_ (TPPO: triphenylphosphine oxide),^22,23^ in which intra-molecular energy transfer occurs from the hfa ligands. We discovered that a host–guest system composed of a 2,4,6-tris(biphenyl-3-yl)-1,3,5-triazine (mT2T) host and the Eu(iii) complex achieves an *I*_PL_ that is three orders of magnitude greater than the *I*_PL_ of the Eu(iii) complex itself and that ∼40 host molecules work for light harvesting of one Eu(iii) complex *via* lossless triplet–triplet inter-molecular energy transfer.

In general, host–guest systems of Ln(iii) complexes have been fabricated as emitting layers in organic light-emitting diodes (OLEDs).^24–26^ Host molecules are known to affect the emission properties of Ln(iii) complexes. Pietraszkiewicz *et al.* fabricated a 5 wt% Eu(nta)_3_SFXPO-complex-doped host–guest film (nta: 1-(2-naphthoyl)-3,3,3-trifluoroacetonate and SFXPO: spiro-fluorene-xanthene diphosphine oxide) with a higher PL quantum yield (PLQY) of 0.86 than 0.64 in a neat film.^27^ This shows that host–guest systems may improve the efficiency of energy transfer processes when compared to molecular Ln(iii) complexes. Buczko *et al.* fabricated a film of 0.17 wt% (N(C_2_H_5_)_4_)[Eu(hfa)_4_]-complexes in (N(C_2_H_5_)_4_)[Ln(hfa)_4_]-complexes, Ln = Gd(iii) or Lu(iii), and found 347 times larger emission intensity compared to the neat film due to inter-molecular energy transfer from ligands of (N(C_2_H_5_)_4_)[Ln(hfa)_4_] and diminishment of concentration quenching.^28^ Nonetheless, there are no design strategies apart from matching the energy of their lowest excited states,^29–33^ and there are few direct observations of inter- and intra-molecular energy transfer processes in host and guest systems.^34–38^

**Fig. 1:**
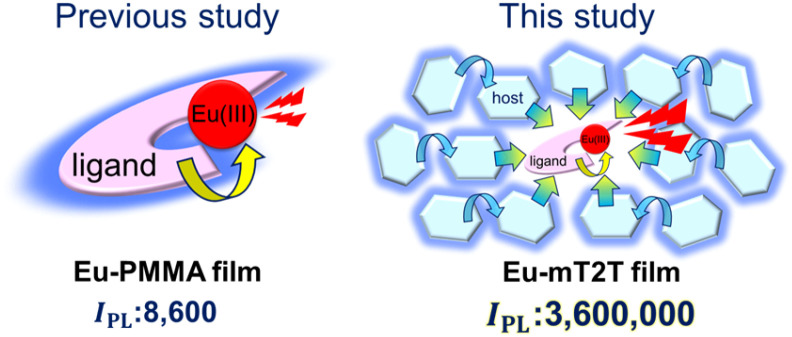
Drastic improvement of the overall photoluminescence intensity (*I*_PL_) by efficient photosensitization from many host molecules. A schematic view of photo-sensitization by intra-molecular energy transfer from the ligands to the Eu(iii) ion in an Eu(hfa)_3_(TPPO)_2_-doped PMMA film (Eu-PMMA film, left) and light harvesting by inter-molecular energy transfer from many hosts to the Eu(iii) complex in the Eu(hfa)_3_(TPPO)_2_-doped mT2T film (Eu-mT2T film, right).

We fabricated 10 wt% Eu(hfa)_3_(TPPO)_2_-doped films with the five host molecules demonstrated below and a polymethyl methacrylate (PMMA) polymer with no photosensitization ability. We measured their *I*_PL_ and discovered that the mT2T host molecule exhibited the highest *I*_PL_ = 3 600 000 M^−1^ cm^−1^, which was approximately four hundred times greater than that of the PMMA host (Fig. 1). To investigate the source of the relatively high *I*_PL_, we used time-resolved PL spectroscopy (TR-PL) and femtosecond transient absorption spectroscopy (fs-TAS) in a multiscale temporal range from sub-picoseconds to hundreds of microseconds to investigate the emission mechanisms of the host–guest film. Beginning with the initial excitation of the host molecules and ending with the emission of the Eu(iii) complex, we elucidated the mechanisms of all processes in the film: (1) the intersystem crossing (ISC) in the host molecule, (2) the inter-molecular energy transfer process from the host molecules to the ligands of the guest Eu(iii) complex, (3) the intra-molecular energy transfer process from the ligands to the Eu(iii) ion, and (4) the emission processes of f–f transitions in the Eu(iii) ion. Furthermore, we discovered that the yields of all energy transfer processes, (1)–(3), were nearly unity and that the yield of the Eu(iii) ion emission process (4) determined the overall quantum yield of the film. This highly efficient PL is attributed to ideal triplet sensitization processes: rapid and efficient ISC in mT2T results in efficient triplet–triplet inter-molecular energy transfers with no losses.

## Results and discussion

### Contribution of host molecules to *I*_PL_

**Fig. 2:**
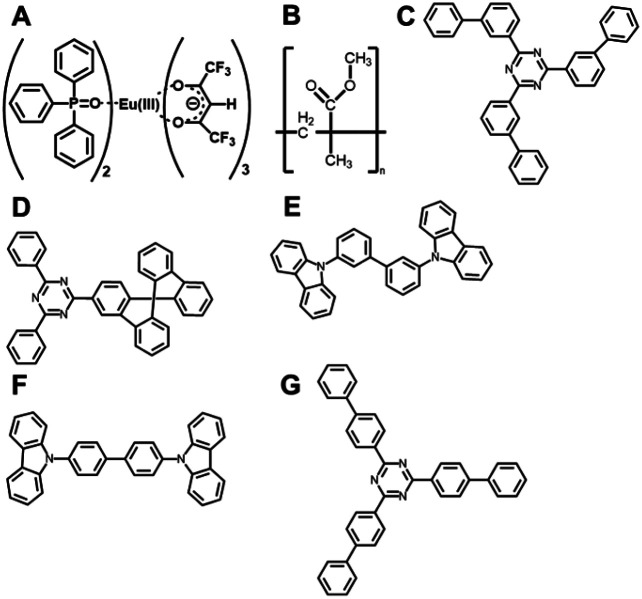
Chemical structures. (A) Eu(hfa)_3_(TPPO)_2_, (B) PMMA, (C) mT2T, (D) SF3TRZ, (E) mCBP, (F) CBP, and (G) T2T.

Five different host molecules were used to fabricate Eu(hfa)_3_(TPPO)_2_-doped films: mT2T, 2-(9,9′-spirobi[fluoren]-3-yl)-4,6-diphenyl-1,3,5-triazine (SF3TRZ), 3,3′-di(9*H*-carbazol-9-yl)biphenyl (mCBP), 4,4′-*N*,*N*′-dicarbazole-biphenyl (CBP), and 2,4,6-tris(1,1′-biphenyl-4-yl)-[1,3,5]triazine (T2T) (Fig. 2 and S2†). These host molecules have been used in conventional OLED applications. To evaluate the intrinsic optical properties of isolated Eu(hfa)_3_(TPPO)_2,_ we also fabricated an Eu(hfa)_3_(TPPO)_2_-doped PMMA (Eu-PMMA) film. Because PMMA is transparent in the >250 nm range,^39^ the absorption spectrum of the Eu-PMMA film in the >250 nm range is identical to that of Eu(hfa)_3_(TPPO)_2_. The absorption range of 250–350 nm for isolated Eu(hfa)_3_(TPPO)_2_ is primarily assigned to the S_0_–S_*n*_ transition of the hfa ligands,^40^ and the absorption of TPPO in this range is much weaker (Fig. S3A†). The host–guest systems significantly enhanced their overall absorption coefficients because all of the host molecules have higher absorption coefficients in the range of 250–280 nm than the hfa ligands (Table 1, Fig. S3B†).

**Table 1:** Absorption and emission properties of the Eu(hfa)_3_(DPPTO)_2_ and Eu(hfa)_3_(TPPO)_2_-doped films. The values of *ε*^mol^_host_ and *ε*_ligands_ of the Eu(hfa)_3_(DPPTO)_2_-PMMA film were evaluated at 267 nm. The values of *ε*_ligands_ of the Eu(hfa)_3_(TPPO)_2_-PMMA film were evaluated at 315 nm. *I*_PL_ was calculated using eqn (1) and (2). The PLQYs (*ϕ*_tot_) were measured at each photoexcitation wavelength (*λ*_ex_) (*ε*_ligands_, *ε*^mol^_host_, *n*_host_, *I*_PL_; see the text for details)

Guest	Host	Eu conc./wt%	*ε*_ligands_/M^−1^ cm^−1^	*ε* ^mol^_host_/M^−1^ cm^−1^	*n*_host_	*ϕ*_tot_	*λ*_ex_/nm	*I*_PL_/M^−1^ cm^−1^
Eu(hfa)_3_(DPPTO)_2_	PMMA	10	1.7 × 10^5^	—	—	0.52	267	8.8 × 10^4^
Eu(hfa)_3_(TPPO)_2_	PMMA	10	1.4 × 10^4^	—	—	0.60	315	8.6 × 10^3^
	mT2T	5	—	1.0 × 10^5^	47	0.76	267	3.6 × 10^6^
		10			22	0.84		1.9 × 10^6^
		30			5.8	0.83		4.8 × 10^5^
		50			2.5	0.78		1.9 × 10^5^
	SF3TRZ	10	—	1.1 × 10^5^	22	0.52	267	1.3 × 10^6^
	mCBP	10	—	4.6 × 10^4^	25	0.30	267	3.4 × 10^5^
	CBP	10	—	4.0 × 10^4^	25	0.24	267	2.1 × 10^5^
	T2T	10	—	1.2 × 10^4^	22	0.22	267	6.1 × 10^4^

**Fig. 3:**
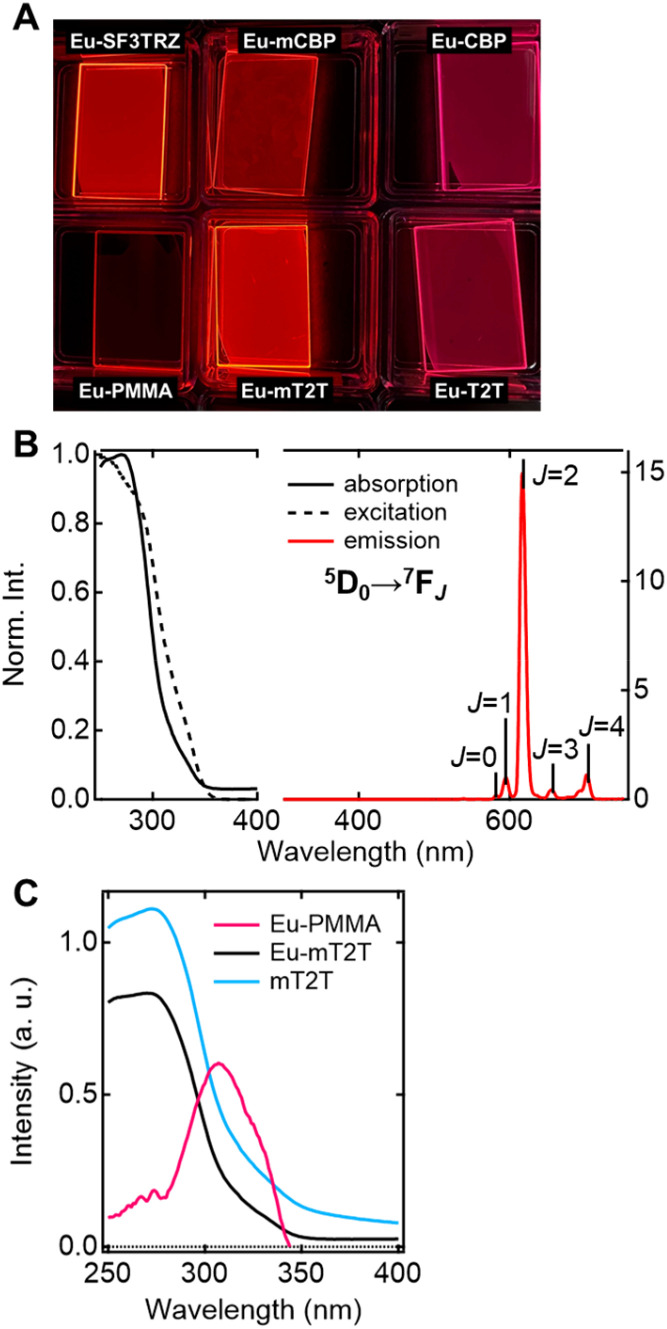
Optical properties of Eu(hfa)_3_(TPPO)_2_-doped films. (A) Photos of emission from the fabricated films upon photoexcitation with 254 nm. (B) Absorption (solid line), excitation probed at 615 nm (broken line), and emission (red line) spectra of the Eu-mT2T film. (C) Absorption spectra of the Eu-PMMA film (pink line), Eu-mT2T film (black line), and mT2T neat film (sky blue line).

To investigate the sensitization ability of the host molecules, we compared the PL properties of the host–guest films to those of the Eu-PMMA film (Fig. 3A). After photoexciting the Eu-PMMA film with 315 nm light, emission bands from the Eu(iii) ion were observed at 581, 594, 615, 654, and 701 nm (Fig. S4†) and are assigned to the transitions ^5^D_0_ → ^7^F_*J*_ and *J* = 0, 1, 2, 3, and 4, respectively.^4^ The Eu(iii) ion sensitization by the hfa ligands in the Eu-PMMA film was confirmed because excitation at 315 nm selectively excites the hfa ligands (Fig. S3A†). Also, after photoexcitation with 260–267 nm light, the same emission bands in the Eu(iii) ion were observed in all of the host–guest films (Fig. 3B and S5†). Given that the absorption coefficients of the host molecules in this wavelength range are much larger than those of the guest complex (Fig. S3†), this indicates that inter-molecular energy transfer from the host molecules to the Eu(iii) complex occurs in all host–guest films.

The sensitization efficiencies of the host–guest films are discussed qualitatively based on their emission spectra. A broad emission band located at around 400 nm was observed for Eu(hfa)_3_(TPPO)_2_-doped SF3TRZ (Eu-SF3TRZ), mCBP (Eu-mCBP), CBP (Eu-CBP), and T2T (Eu-T2T) films, in addition to the emission from the Eu(iii) ion (Fig. S5†). These bands were assigned to the fluorescence from the lowest singlet excited state (S_1_) of each host molecule (Fig. S6†), indicating imperfect energy transfer to the Eu(iii) complex. In contrast, no emission band of the host molecule was observed in the Eu(hfa)_3_(TPPO)_2_-doped mT2T (Eu-mT2T) film (Fig. 3B). This finding suggests that inter-molecular energy transfer from the host mT2T molecules to the Eu(iii) complex occurs extremely efficiently in the film. In fact, *ϕ*_tot_ of Eu-mT2T (*ϕ*_tot_ = 0.84) was significantly higher than that of Eu-PMMA (*ϕ*_tot_ = 0.60) (Table 1). The excitation spectra probed at the Eu(iii) ion emission, which coincided with the absorption spectra of these films, further supported efficient sensitization (Fig. 3B and C).

We measured the photophysical properties of Eu-mT2T films with different doping ratios of the Eu(iii) complex to mT2T to estimate the number of host molecules that contributed to one Eu(iii) ion emission (Table 1). Considering this result, *I*_PL_ of host–guest films can be expressed as

**Fig. 4:**
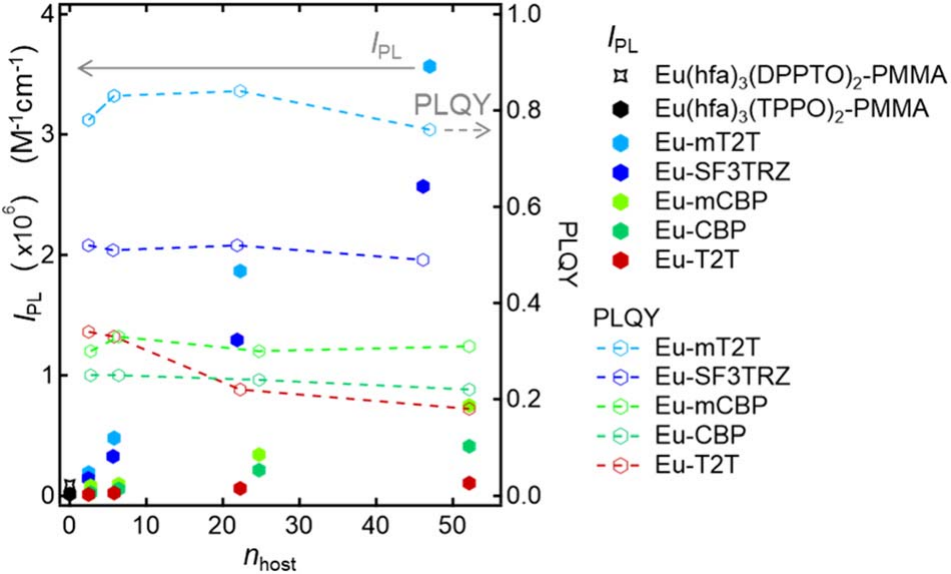
Host and its ratio dependence of overall luminescence intensities (*I*_PL_) and PLQY. *I*_PL_ of the Eu(hfa)_3_(DPPTO)_2_-doped PMMA film (black star marker), Eu(hfa)_3_(TPPO)_2_-doped PMMA film (black hexagon marker), Eu-mT2T film (sky blue hexagon markers), Eu-SF3TRZ film (blue hexagon marker), Eu-mCBP film (yellow green hexagon marker), and Eu-T2T film (red hexagon marker), calculated using eqn (1) and (2). PLQY of the Eu-mT2T film (sky blue line), Eu-SF3TRZ film (blue line), Eu-mCBP film (yellow green line), and Eu-T2T film (red line). *n*_host_ is the number of host molecules that contributed to one Eu(iii) ion emission.

2 *I*_PL_ = *ε*_hosts_ × *ϕ*_tot_ = *ε*^mol^_host_ × *n*_host_ × *ϕ*_tot_

where *ε*^mol^_host_ and *n*_host_ represent the molar absorption coefficient of the host molecule and the number of host molecules that contributed to one Eu(iii) ion emission which is calculated from the doped molar ratio between the host molecule and the Eu(iii) complex, respectively. The *ϕ*_tot_ was the highest when the mixing ratio was 10%, implying that ∼22 host molecules contributed to the emission of one Eu(iii) complex *via* energy transfer between the host and guest molecules. The *I*_PL_ is plotted as a function of *n*_host_ in Fig. 4. The *I*_PL_ for the Eu(hfa)_3_(TPPO)_2_-doped films increases monotonically even when *n*_host_ = 47, indicating that more than 47 host molecules work as photosensitizers for one Eu(iii) complex. Note that the maximum *I*_PL_ is more than two orders of magnitude greater than that of the conventional highly luminescent complex Eu(hfa)_3_(DPPTO)_2_.^11^

### Initial process after photoexcitation: rapid and efficient ISC in mT2T

**Fig. 5:**
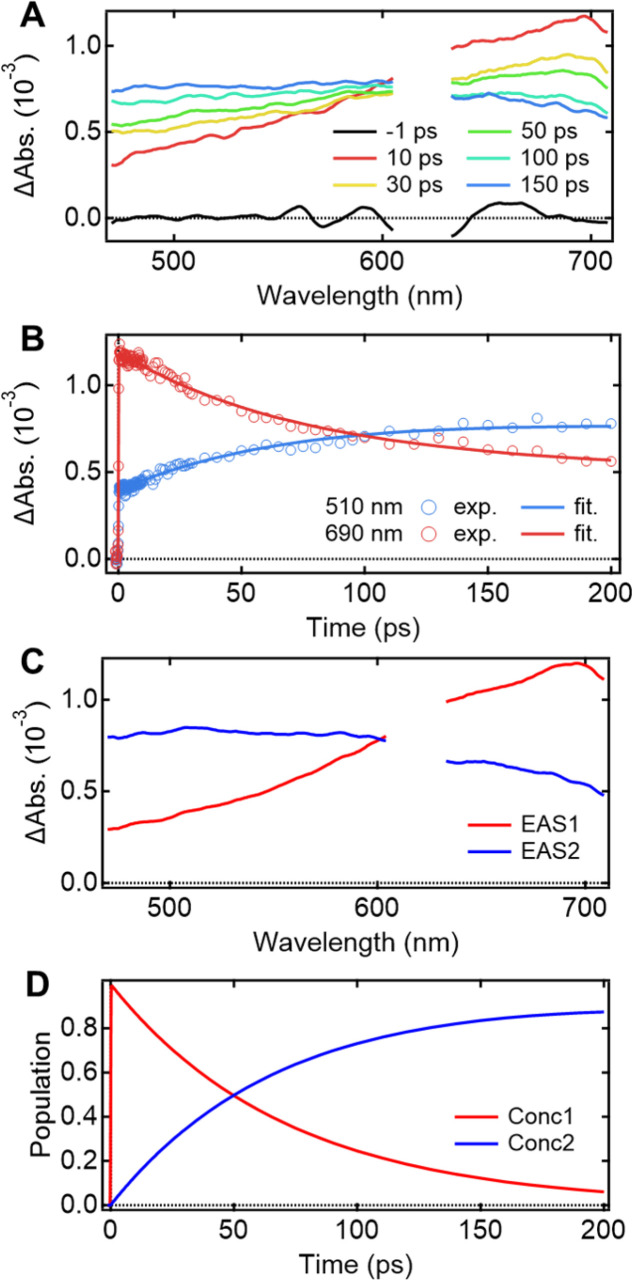
fs-TAS spectra and the results of their global analysis for the Eu-mT2T film. (A) Temporal evolutions of the fs-TAS spectra after photoexcitation at 267 nm. (B) Temporal profiles of the fs-TAS spectra at 510 nm (blue circles) and 690 nm (red circles) and the corresponding fitting curves which resulted from the global analysis. (C) EAS. (D) Corresponding concentration kinetics obtained from the global analysis. No TA data were available due to the strong emission from the Eu(iii) ion in the region neighboring 615 nm.

Understanding the mechanisms of intra- and inter-molecular energy transfer processes requires understanding the photophysical processes occurring in host molecules. To investigate the processes in the time domain, we measured and compared the fs-TAS spectra of the Eu-mT2T film (Fig. 5A) and the mT2T neat film (Fig. S7A†). The absorbance change (ΔAbs.) of the Eu-mT2T film increased in the <600 nm range, whereas it decreased in the >600 nm range, with an isosbestic point at 600 nm. We note that the isosbestic point does not appear as an intersection at one point due to a slight spectral shift over time. This is presumably due to the dynamical effect uniquely seen in a solid state, for example, dielectric relaxation. We performed a global analysis of the fs-TAS spectra, assuming a sequential model with two components because the isosbestic point indicates an exclusive transition between two states.^41^ With a time constant of 71.2 ± 0.6 ps, the first component was converted to the second component (Fig. 5B). The evolution associated spectra (EAS) and concentration kinetics of the two components are shown in Fig. 5C and D, respectively. We analyzed the fs-TAS of the mT2T neat film in the same way to assign the observed species. The global analysis also resulted in similar EASs (Fig. S7C†), with a time constant of 44.9 ± 0.4 ps (Fig. S7D†). The two EAS components were reasonably assigned to S_1_ and the lowest triplet excited state (T_1_) in the case of the neat film, and the time constant represented the ISC rate. Therefore, the two components observed in the Eu-mT2T film were assigned to S_1_ and T_1_ of the host mT2T molecule. These time constants are much faster than those of the ISC process for aromatic organic molecules in general, which can be explained by the presence of lone pairs in the triazine moieties in mT2T accelerating the ISC rate.^42,43^ We conclude that mT2T undergoes a rapid and nearly unity ISC before exciton diffusion and energy transfer in the host–guest film.

### Quantum yields of each energy transfer process

**Fig. 6:**
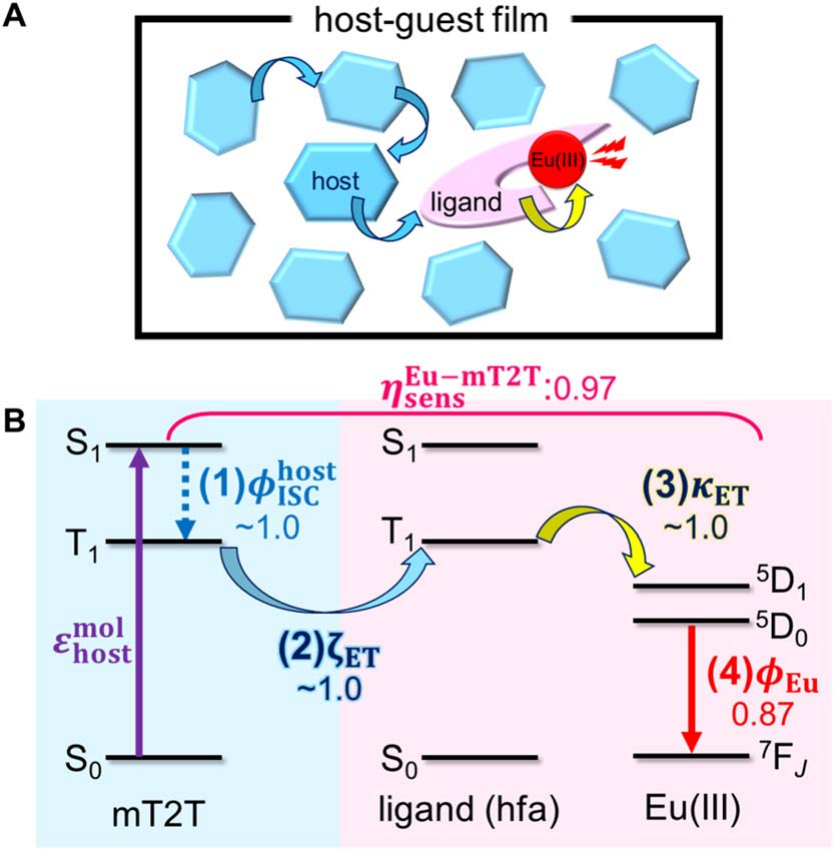
Overview of inter- and intra-molecular energy transfer processes in the Eu-mT2T film. (A) Schematic in real space. (B) Schematic of energy levels. *ϕ*^host^_ISC_, *ζ*_ET_, *κ*_ET_, and *ϕ*_Eu_ represent the quantum yields of (1) ISC in mT2T, (2) inter-molecular energy transfer from mT2T to the hfa ligands, (3) intra-molecular energy transfer from the hfa ligands to the Eu(iii) ion, and (4) the emission in the Eu(iii) ion, respectively. *η*^Eu-mT2T^_sens_ is the overall photosensitization efficiency in the Eu-mT2T film (eqn (4)).

Given that rapid ISC occurs first in the host film, Fig. 6 shows the predicted energy transfer processes in the Eu-mT2T film after photoexcitation. We first estimated the luminescence quantum yield of the Eu(iii) ion (*ϕ*_Eu_) in the host–guest films to estimate the quantum yield of each process. The natural radiative rate constant of the Eu(iii) ion (*k*^Eu^_r_) can be calculated from the ratio of the total Eu(iii) ion transition to the magnetic dipole transition (^5^D_0_ → ^7^F_1_) in the observed emission spectrum of the Eu(iii) ion (Fig. 3B, eqn (S1); see the ESI text for details†).^44^*ϕ*_Eu_ is also determined using the ratio of *k*^Eu^_r_ to the observed decay rate constant of the ^5^D_0_ state (*k*^Eu^_obs_) (eqn (S2) and (S3)†). The estimated *ϕ*_Eu_, rate constants, and parameters are summarized in Table S1.† We calculated *η*_sens_ because *ϕ*_tot_ represents the product of *ϕ*_Eu_ and overall photosensitization efficiency (*η*_sens_; eqn (S4); Table S1†). It is worth noting that *η*_sens_ in the 10 wt% Eu-mT2T film is nearly unity, with *η*^Eu-mT2T^_sens_ = 0.97. This indicates approximately perfect sensitization of photoexcited mT2T to the Eu(iii) complex in the Eu-mT2T film, which is more efficient than sensitization of hfa ligands in the Eu-PMMA film, *η*^Eu-PMMA^_sens_ = 0.71.

To identify the factor that improves energy transfer efficiency in the Eu-mT2T film, we compared the sensitization efficiencies of intra-molecular energy transfer in the Eu-PMMA film (*η*^Eu-PMMA^_sens_) and the inter- and intra-molecular energy transfer in the Eu-mT2T film (*η*^Eu-mT2T^_sens_). *η*^Eu-PMMA^_sens_ is expressed as follows:

3 *η*^Eu-PMMA^_sens_ = *ϕ*^ligand^_ISC_ × *κ*_ET_

where *ϕ*^ligand^_ISC_ and *κ*_ET_ represent the ISC yield of the ligand and the efficiency of intra-molecular energy transfer, respectively. In contrast, *η*^Eu-mT2T^_sens_is expressed as

4 *η*^Eu-mT2T^_sens_ = *ϕ*^host^_ISC_ × *ζ*_ET_ × *κ*_ET_

where *ϕ*^host^_ISC_and *ζ*_ET_ represent the yield of ISC of the host and the efficiency of inter-molecular energy transfer, respectively. Fig. 6B shows the symbols for the Eu-mT2T film. We conducted time-resolved measurements in Eu(hfa)_3_(TPPO)_2_-doped neat (Eu-neat) and Gd(hfa)_3_(TPPO)_2_-doped neat (Gd-neat) films to estimate the *κ*_ET_ of the Eu(iii) complex (Fig. S8 and S9†). We can observe intrinsic emission from the hfa ligands in the Gd-neat film because there is no intra-molecular energy transfer to Gd(iii) due to the energy level mismatch (Fig. S9A–C†). We compared the time constants of the fs-TAS and TR-PL measurements to analyze each time constant in the energy transfer process (Fig. S10†). The *κ*_ET_ of the Eu(iii) complex was >0.99 when the lifetimes of the T_1_ ligands between the Eu-neat film and the Gd-neat film were compared (Table S2, eqn (S5)†). We conclude that the quantum yield of the ISC at the ligands, *ϕ*^ligand^_ISC_, in the Eu-PMMA film is the dominant factor in the relatively low *η*^Eu-PMMA^_sens_. Sensitization processes in the case of the Eu-mT2T film, in contrast, occur *via* inter-molecular energy transfer *via* T_1_ states between the mT2T and hfa ligands. Because the quantum yield of ISC in mT2T is nearly unity, the Eu-T2T film achieves more efficient sensitization.

### Time-domain view of the whole sensitization processes

**Fig. 7:**
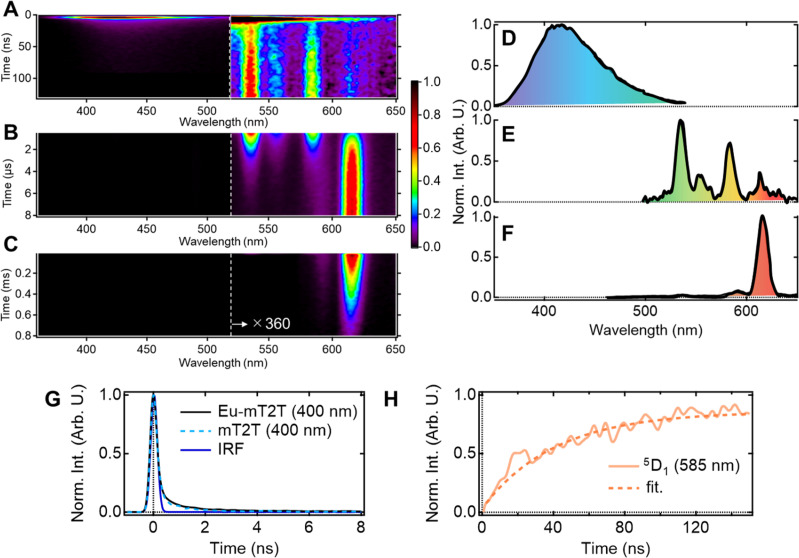
TR-PL spectra and their temporal profiles. (A–C) Pseudo-2D plot of the emission from the Eu-mT2T film after photoexcitation at 267 nm in the time range of (A) 0–130 ns, (B) 0.5–8 μs, and (C) 0.01–0.8 ms. For clarity, the intensities above 520 nm are magnified by a factor of 360. (D–F) Normalized emission intensity spectra were obtained from the streak images by the time integration of (D) 0.05–0.15 ns, (E) 145–155 ns, and (F) 6.5–7.5 μs. (G) Temporal profiles of the emission at 400 nm after photoexcitation of the Eu-mT2T film (solid black line) and the mT2T neat film (broken sky blue line) together with the instrumental response function (IRF, solid blue line). (H) Temporal profiles of the emission at 585 nm subtracted from the overlapped fluorescence of the host molecules after photoexcitation of the Eu-mT2T film (orange line) and fitting curve (broken orange line).

Multiscale TR-PL measurements were performed to quantify energy transfer processes in the time domain. The pseudo-2D color plot of the TR-PL of the Eu-mT2T film after photoexcitation at 267 nm is shown in Fig. 7A–C. Following photoexcitation, a broad emission band in the 350–500 nm range was observed (Fig. 7A and D). This band shape is approximately identical to that of the fluorescence observed in the mT2T neat film (Fig. S11†); this emission is attributed to the S_1_ emission in mT2T. Note that no emission from mT2T was observed in the steady-state emission spectrum (Fig. 3B) because its time-integrated intensity was much smaller than that of the Eu(iii) ion. The decay time constant of mT2T emission was estimated to be <100 ps (Fig. 7G), which agrees with the time constant of ISC in mT2T (∼70 ps) estimated from fs-TAS spectra.

Following the rapid decay of fluorescence from mT2T, narrow-band emissions in the nanosecond to microsecond time range were observed (Fig. 7B, C, E and F). According to Dicke's diagram, all of these emission bands were assigned to the f–f transitions in the Eu(iii) ion.^45^ The 535, 555, and 585 nm bands in the nanosecond region (Fig. 7A and E) were assigned to the transitions ^5^D_1_ → ^7^F_1_, ^5^D_1_ → ^7^F_2_, and ^5^D_1_ → ^7^F_3_, respectively. The bands at 590 and 615 nm in the microsecond region (Fig. 7B and F) were assigned to ^5^D_0_ → ^7^F_1_ and ^5^D_0_ → ^7^F_2_, respectively. The rise time constant of the ^5^D_1_ state was estimated to be 41.0 ± 0.8 ns (Fig. 7H), which is much longer than the time constant of the ISC in mT2T (<100 ps). The direct energy transfer from the T_1_ state in mT2T to the excited states in the Eu(iii) ion is ruled out because this energy transfer occurs by the Dexter mechanism with electron exchange and the distance between mT2T and the Eu(iii) ion in the film is too long for such an energy transfer to occur.

Therefore, this result indicates that the energy transfer to the Eu(iii) complex is ∼40 ns and is mediated *via* the T_1_ states. The rise time is determined by multiple processes: (1) the ISC of mT2T (<100 ps), (2) the triplet exciton diffusion in the host matrix, (3) the triplet–triplet energy transfer between mT2T and the hfa ligand, and (4) the intramolecular energy transfer from the hfa ligand to the Eu(iii) ion.

### Mechanisms of efficient triplet-state mediated energy transfer and sensitization

Here, we discuss the origins of efficient inter-molecular energy transfer in the Eu-mT2T film in terms of T_1_ energy matching. We compared the T_1_ energies of the mT2T molecule to those of the hfa ligands of the Eu(iii) complex. The T_1_ energies for mT2T and hfa estimated from the phosphorescence spectra (Fig. S12†) were found to be 2.66 and 2.70 eV, respectively. Due to the close proximity of the energies, an efficient energy transfer from mT2T to the Eu(iii) complex is anticipated. We also compared the T_1_ energies of the other host molecules to those of hfa, confirming that energetically resonant conditions in the T_1_ energies of the host and hfa are important for the higher PLQYs in the host–guest films (Fig. S12G†). This indicates that inter-molecular energy transfer from host molecules to the Eu(iii) ion occurs *via* the T_1_ of hfa. Furthermore, the phosphorescence of hfa ligands was observed following energy transfer from T_1_ in mT2T (Fig. S12A and S13†) in the Gd(hfa)_3_(TPPO)_2_-doped mT2T (Gd-mT2T) film, consistent with efficient host-to-hfa energy transfer based on triplet–triplet energy transfer. We concluded that the energy resonance in T_1_ between mT2T and hfa causes highly efficient inter-molecular energy transfer sensitization (*ζ*_ET_ ∼ 1.0).

It is worth mentioning that our triplet-based emission enhancement strategy offers several advantages over the co-fluorescence effect, in which a Gd or Lu complex works as a sensitizer, as described in ref. 28. Firstly, we achieved a significantly greater enhancement in emission compared to the previous work. Secondly, we utilized organic compounds as host molecules, resulting in a substantial improvement in absorption ability. Thirdly, the host–guest films are fabricated through a simple solution process.

There are two possible assignments to the slow rise time. One is the energy transfer from the T_1_ of mT2T to the T_1_ of the ligand and the other is the triplet exciton diffusion in the host matrix. Finally, we discuss triplet exciton diffusion in the host matrix by comparing energy transfer to the Eu(iii) complex and PLQYs in Eu-mT2T films with different mT2T and Eu(iii) complex ratios (Fig. S14, Table S3†). As the concentration of the Eu(iii) complex increased, the rise time constant of ^5^D_1_ got shorter (Fig. S14†). The rise time constant of the low ratio of the mT2T (Eu(iii) complex concentration of 50 wt%) film exhibits a fastest rise time constant (∼29 ns). Because the lifetime of T_1_ in mT2T is likely to be much longer than the diffusion time scale, sensitization processes mediated by triplet–triplet energy transfer are effective in realizing ideal sensitization for Eu(iii) ion emission (Fig. S15†).

## Conclusions

We demonstrated highly efficient light harvesting of the Eu(iii) ion in complex-doped host–guest films with Eu(hfa)_3_(TPPO)_2_. When we used mT2T, a triazine derivative that works well as an energy-harvesting antenna, we observed a significant increase in the luminescence intensity of the Eu(iii) ion. From the photoexcitation of host molecules to the emission of Eu(iii) ion, we estimated the quantum yields of all energy transfer processes and discovered that all energy transfer processes occur with nearly unity quantum yield. We conclude from the TR-PL and fs-TAS measurements that efficient energy transfer occurs *via* resonant energy transfer from T_1_ of mT2T to T_1_ of hfa following rapid and highly efficient ISC in mT2T. This mechanism can avoid energy loss in the ISC process in the hfa ligands of an Eu(iii) complex and overcome the intrinsic limitations of conventional direct sensitization by the ligands. Based on these results, we propose a novel light harvesting method for Ln(iii) with simple fabrication: a host–guest film composed of host molecules with efficient ISC, which works as an efficient photosensitizer, and a guest Ln(iii) complex with ligands having a T_1_ state whose energy matches that of T_1_ in the host, which works as an efficient energy accepter and emitter.

## Experimental

### Materials

A previously reported procedure was used to synthesize Eu(hfa)_3_(TPPO)_2_.^22^ mT2T, SF3TRZ, and mCBP were purchased from the NARD Institute Ltd. (Hyogo, Japan). CBP was purchased from Angene International Ltd. (Nanjing, China). T2T was purchased from Tokyo Chemical Industry Co., Ltd. (Tokyo, Japan). mT2T, SF3TRZ, mCBP, and CBP were purified *via* sublimation. Chloroform, dichloromethane, and methanol were purchased from Kanto Kagaku Co., Ltd. (Tokyo, Japan). Without further purification, all solvents were used as received.

### Fabrication of thin films

Thin films for optical measurements were fabricated by spin-coating on quartz substrates. The quartz substrates were washed by ultrasonic cleaning with acetone and isopropanol. For the preparation of neat films, the emitter compounds were dissolved in chloroform (10 wt%). To prepare the host–guest films, a weight ratio of 1 : 9 of the guest molecule and the host molecule was dissolved in chloroform to obtain an overall concentration of 10 wt%. Before use, the solution was filtered through a 0.2 μm filter, and the quartz substrates were heated to 80 °C. The solution was spin-coated onto quartz substrates for 60 s at 1000 rpm and then annealed at 70 °C for 10 min. Table S4† shows the thicknesses of the films. Thin films for refractive index measurements were fabricated on silicon substrates using vacuum vapor deposition at a pressure of less than 10^−3^ Pa. We used a fixed deposition rate of 0.5 nm s^−1^ and a thickness of 100 nm.

### General methods

UV-vis absorption spectra were measured using a PerkinElmer LAMBDA 950 spectrophotometer. Excitation and PL spectra of the Eu(hfa)_3_(TPPO)_2_-doped PMMA film (excitation wavelength for PL spectra: *λ*_ex_ = 315 nm and probe wavelength for excitation spectra: *λ*_em_ = 615 nm), Eu(hfa)_3_(TPPO)_2_-doped mT2T film (*λ*_ex_ = 267 nm and *λ*_em_ = 615 nm), Eu(hfa)_3_(TPPO)_2_-doped T2T film (*λ*_ex_ = 260 nm and *λ*_em_ = 615 nm), Eu(hfa)_3_(TPPO)_2_-doped SF3TRZ film (*λ*_ex_ = 267 nm and *λ*_em_ = 615 nm), Eu(hfa)_3_(TPPO)_2_-doped mCBP film (*λ*_ex_ = 267 nm and *λ*_em_ = 615 nm), and Eu(hfa)_3_(TPPO)_2_-doped CBP film (*λ*_ex_ = 260 nm and *λ*_em_ = 615 nm) were measured using spectrofluorometers (FP-8600, JASCO; PMA-12, Hamamatsu Photonics). The phosphorescence spectra of the neat films of Gd(hfa)_3_(TPPO)_2_, TPPO (*λ*_ex_ = 335 nm), Gd(hfa)_3_(TPPO)_2_-doped mT2T (*λ*_ex_ = 267 nm), mT2T (*λ*_ex_ = 267 nm), T2T (*λ*_ex_ = 315 nm), SF3TRZ (*λ*_ex_ = 267 nm), mCBP (*λ*_ex_ = 267 nm), and T2T (*λ*_ex_ = 267 nm) at 77 K were measured using a spectrofluorometer (FP-8600, JASCO; PMA-12, Hamamatsu Photonics).

The photoluminescence quantum yields were measured using a Hamamatsu Photonics Quantaurus-QY instrument equipped with an integrating sphere. A time-correlated single-photon counting lifetime spectroscopy system (HAMAMATSU Quantaurus-Tau C11367-21, C11567-02, and M12977-01) was used to measure PL lifetimes.

The refractive indexes and thicknesses of the films were measured using variable-angle spectroscopic ellipsometry (M-2000U, J. A. Woollam Co., Inc., United States).

### Time-resolved photoluminescence (TR-PL)

TR-PL measurements were performed using a streak camera system (Hamamatsu C4780, time resolution < 30 ps) synchronized with a Ti:sapphire regenerative amplifier (Spectra-Physics, Spitfire Ace, pulse duration = 120 fs, repetition rate = 1 kHz, pulse energy = 4 mJ per pulse, central wavelength = 800 nm).^34^ The samples were excited by the third harmonic (267 nm) of the fundamental pulse from the amplifier. Before measuring, all films were encapsulated. The excitation energy was kept to less than 0.8 mJ cm^−2^.

### Femtosecond transient absorption spectroscopy (fs-TAS)

Transient absorption (TA) measurements were conducted using the pump–probe method.^46^ The light source was a Ti:sapphire regenerative amplifier system (Spectra-Physics, Spitfire Ace, pulse duration = 120 fs, repetition rate = 1 kHz, pulse energy = 4 mJ per pulse, central wavelength = 800 nm) seeded using a Ti:sapphire femtosecond mode-locked laser (Spectra-Physics, Tsunami). The output of the amplifier was divided into two pulses for the pump and probe. The samples were pumped by the third harmonic of the fundamental pulse from an amplifier (267 nm). The broadband probe pulse (450–750 nm) was generated using a sapphire crystal of 1 mm thickness. The pump and probe pulse beam sizes at the sample position were <0.7 mm*ϕ* and <0.5 mm*ϕ*, respectively. A PC-controlled mechanical delay state was used to adjust the delay time between the pump and probe pulses. The probe pulse that passed through the sample films was dispersed using a polychromator (JASCO, CT-10, 300 grooves/500 nm), and the spectra were captured using a multichannel detection system with a CMOS sensor (UNISOKU, USP-PSMM-NP). To avoid damage, all the films were encapsulated before being measured and were mechanically moved continuously. The excitation energy was kept to less than 0.4 mJ cm^−2^. The recorded data were analyzed using a Python-based homemade program. Note that the Eu-mT2T film shows much stronger emission compared to general Eu(iii) complexes, so that no TA data were available at the positions of the Eu(iii) ion emission.

## Author contributions

SM prepared the films. YK synthesized the complexes. KG and SM measured the optical properties of the sample. SM conducted the time-resolved spectroscopic measurements. KG and SM analyzed all the data. KO, KM, and SM drafted the original manuscript. KO and KM supervised the study. All authors contributed to the review and editing of the manuscript and critically commented on the project.

## Conflicts of interest

There are no conflicts to declare.

## Supplementary Material

SC-014-D3SC01817B-s001
