# Supplementary material for: Highly efficient light harvesting of a Eu(iii) complex in a host–guest film by triplet sensitization
Source: Chem Sci. 2023 May 29;14(25):6867–75. doi: 10.1039/d3sc01817b (PMC10306081; doi:10.1039/d3sc01817b)
Supplement: SC-014-D3SC01817B-s001 [file SC-014-D3SC01817B-s001.pdf]

Electronic Supplementary Material (ESI) for Chemical Science.
This journal is © The Royal Society of Chemistry 2023

# Supporting Information for

## Highly Efficient Light Harvesting of Eu(III) Complex in a Host-Guest Film by Triplet Photosensitization

Shiori Miyazaki, [1] Kenichi Goushi,[2,3] Yuichi Kitagawa,[4,5] Yasuchika Hasegawa,[4,5] Chihaya Adachi,[2,3] Kiyoshi Miyata,[1]* and Ken Onda[1]*

[1] Department of Chemistry, Kyushu University, 744 Motooka, Nishi, Fukuoka 819-0395, Japan.

[2] Center for Organic Photonics and Electronics Research (OPERA), Kyushu University, 744 Motooka, Nishi, Fukuoka 819-0395, Japan.

[3] International Institute for Carbon Neutral Energy Research (I2CNER), Kyushu University, 744 Motooka, Nishi, Fukuoka 819-0395, Japan.

[4] Faculty of Engineering, Hokkaido University, N13W8, Kita-ku, Sapporo, Hokkaido 060-8628, Japan.

[5] Institute for Chemical Reaction Design and Discovery (WPI-ICReDD), Hokkaido University, N21 W10, Kita-ku, Sapporo, Hokkaido 001-0021, Japan.

*Corresponding author. E-mail: kmiyata@chem.kyushu-univ.jp (K.M.); konda@chem.kyushu-univ.jp (K.O.)

## Supplementary Text

Calculation of the luminescence quantum yield (*1*)

The transitions $^5D_0 \rightarrow ^7F_{0,\,2,\,3,\,4}$ are electric dipole transitions whose intensities are increased by reducing the coordination symmetry of the Eu(III) complex. The $^5D_0 \rightarrow ^7F_1$ transition, in contrast, is a magnetic dipole transition that is independent of the coordination structure. We can assume that the energy of the $^5D_0 \rightarrow ^7F_1$ transition and the dipole strength are constant. The relationship between the shape of the emission spectrum of Eu(III) ion and its radiative rate constant ($k_r^{Eu}$) is expressed as

$$k_r^{Eu} = A_{MD,0} n^3 (\frac{I_{tot}}{I_{MD}}) \tag{S1}$$

Here, $A_{MD,0}$, $n$, and ($I_{tot}/I_{MD}$) represent the spontaneous luminescence rate of the $^5D_0 \rightarrow ^7F_1$ transition *in vacuo* (14.65 $s^{-1}$), refractive index of the medium, and ratio of the total area of the Eu(III) luminescence spectrum to the area of the $^5D_0 \rightarrow ^7F_1$ transition band, respectively.

The observed decay rate constant ($k_{obs}^{Eu}$) of the $^5D_0$ state is expressed as

$$k_{obs}^{Eu} = \frac{1}{\tau_{obs}^{Eu}} = k_r^{Eu} + k_{nr}^{Eu} \tag{S2}$$

Here, $\tau_{obs}^{Eu}$, $k_r^{Eu}$, and $k_{nr}^{Eu}$ represent the observed lifetimes of the $^5D_0$ state, the radiative rate constant of Eu(III) ion, and the non-radiative rate constant of Eu(III) ion, respectively. The quantum yield of Eu(III) luminescence ($\phi_{Eu}$) and overall photosensitization efficiency ($\eta_{sens}$) were determined as follows:

$$\phi_{Eu} = \frac{k_r^{Eu}}{k_r^{Eu}+k_{nr}^{Eu}} = \frac{k_r^{Eu}}{k_{obs}^{Eu}} \tag{S3}$$

$$\phi_{tot} = \eta_{sens} \times \phi_{Eu} \tag{S4}$$

Calculation of the intra-molecular energy transfer efficiency ($\kappa_{ET}$)

The intra-molecular energy transfer efficiency from the ligands to the Eu(III) ion ($\kappa_{ET}$) was determined under the assumption that the radiative and non-radiative decay rates of the triplet state for the Gd(III) complexes reflect those of the Eu(III) complexes in the absence of energy transfer such that

$$\kappa_{ET} = 1 - \frac{k_{Gd}^{T_1}}{k_{Eu}^{T_1}} \tag{S5}$$

where $k_{\mathrm{Gd}}^{\mathrm{T1}}$ and $k_{\mathrm{Eu}}^{\mathrm{T1}}$ represent the rate constants of the $T_1$ states of the Gd (III) complex and the ligands in the Eu (III) complex ligands, respectively. In this case, it is assumed that there is no energy loss process from the $T_1$ states of the ligands to other energy states such as the ligand-to-metal charge transfer (LMCT) states (*2, 3*). This assumption is justified because (1) the energy levels of the HOMOs in the ligands in the Eu(III) complexes are too low to provide LMCT state in the visible energy region and (2) no band originating from LMCT state was observed in the absorption spectrum of the Eu(III) complexes we investigated in this work.

## Figures and Tables

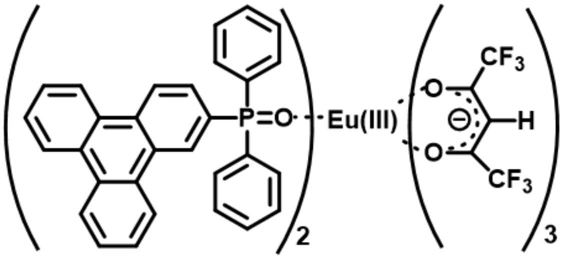


**Fig. S1. Chemical structure of Eu(hfa)$_3$(DPPTO)$_2$.**

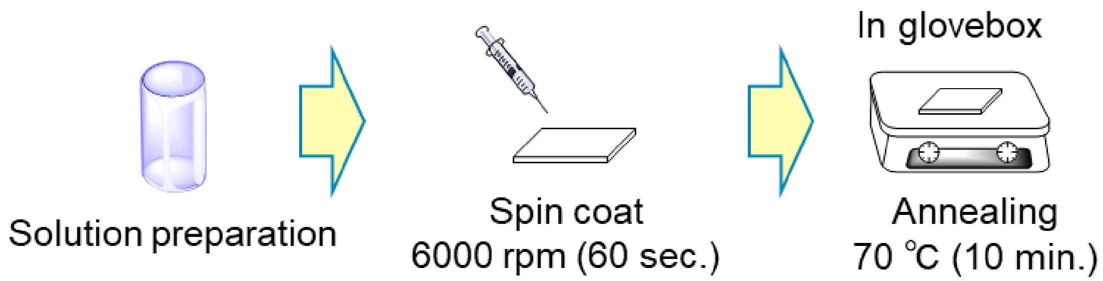


**Fig. S2. Fabrication process of the host-guest film.**

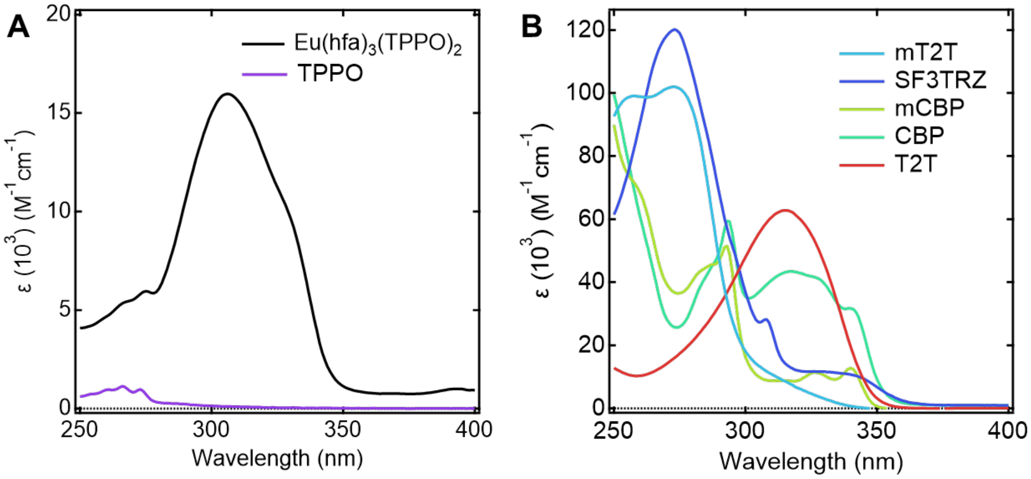


**Fig. S3. Absorption spectra. (A)** Absorption spectra of $Eu(hfa)_3(TPPO)_2$ (black line) and TPPO (purple line). **(B)** Absorption spectra of mT2T (sky blue line), SF3TRZ (blue line), mCBP (yellow green line), CBP (green line), and T2T (red line). The spectrum of $Eu(hfa)_3(TPPO)_2$ was measured in $CHCl_3$. The spectra of TPPO and all host molecules were measured in $CH_2Cl_2$.

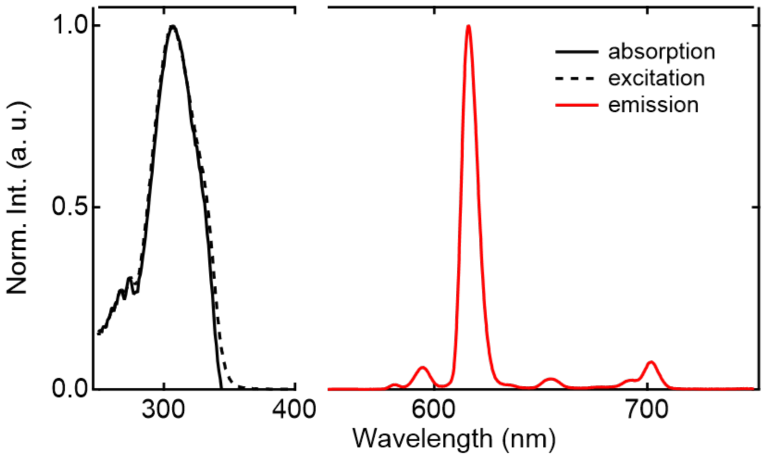


**Fig. S4. Optical spectra of the Eu-PMMA film.** Absorption (solid line), excitation (broken line, probed at 615 nm), and emission (red line, $\lambda_{ex}$ = 315 nm) spectra of the Eu-PMMA film. The spectral shapes of the absorption and excitation spectra were almost identical, indicating intra-molecular energy transfer from the hfa ligands to the Eu(III) ion.

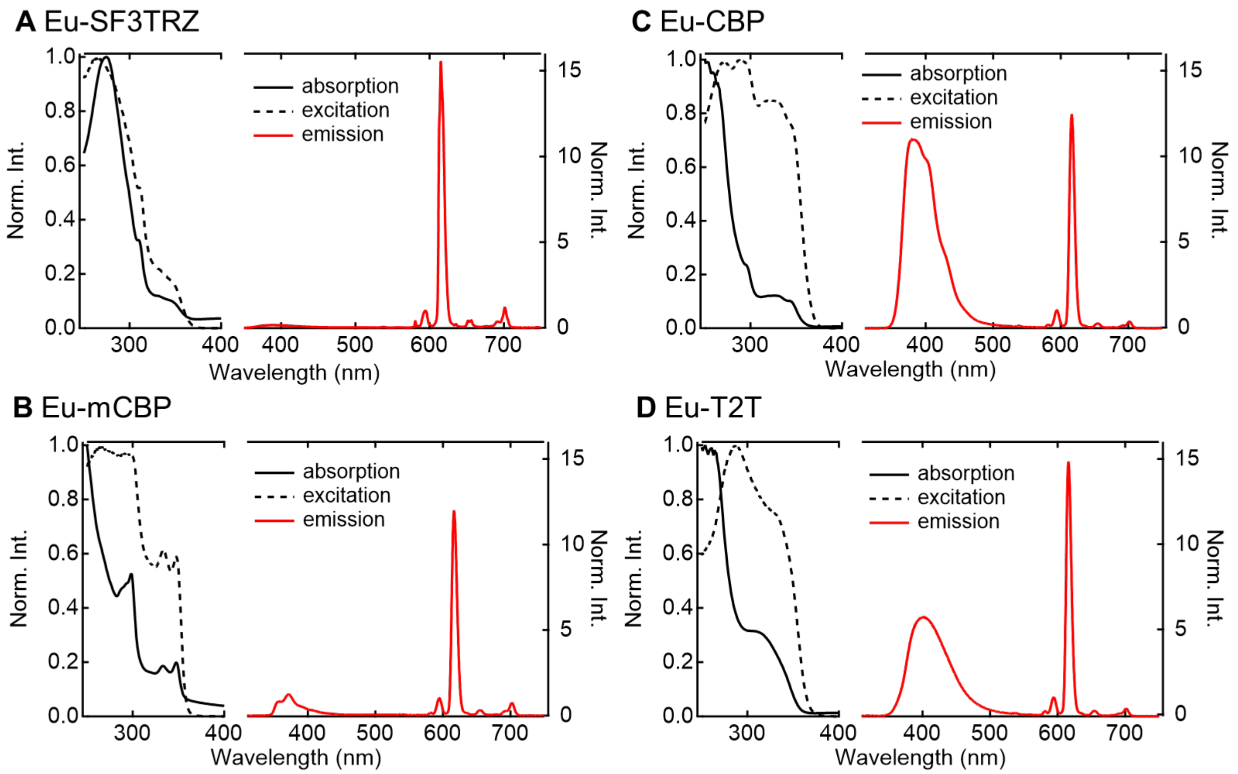


**Fig. S5. Optical spectra of the host-guest films.** Absorption (black line), excitation (broken line, probed at 615 nm), and emission spectra (red line) of the **(A)** Eu-SF3TRZ film ($\lambda_{ex}$ = 267 nm), **(B)** Eu-mCBP film ($\lambda_{ex}$ = 267 nm), **(C)** Eu-CBP film ($\lambda_{ex}$ = 260 nm), and **(D)** Eu-T2T film ($\lambda_{ex}$ = 260 nm).

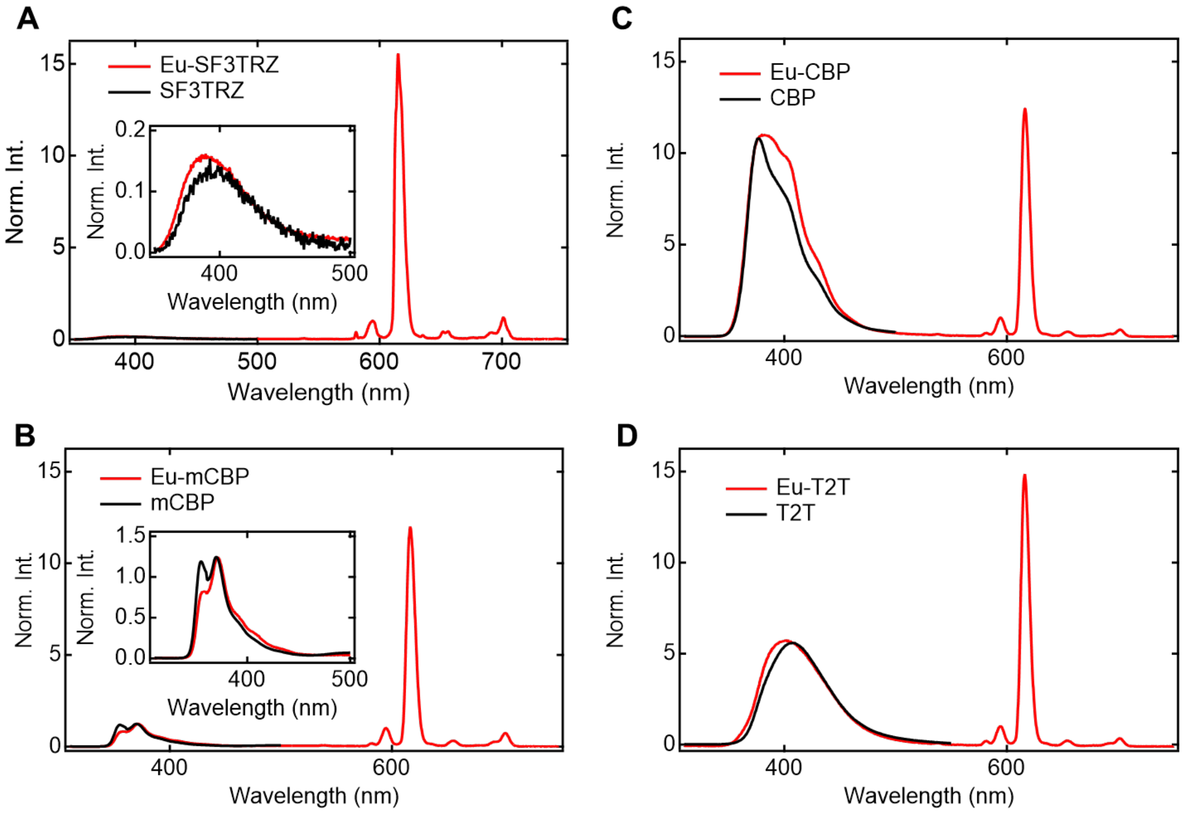


**Fig. S6. Comparison of emission spectra between host-guest and host-molecule neat films.** **(A)** Eu-SF3TRZ film (red line, $\lambda_{ex}$ = 267 nm) and SF3TRZ neat film (black line, $\lambda_{ex}$ = 275 nm). **(B)** Eu-mCBP film (red line, $\lambda_{ex}$ = 267 nm) and mCBP neat film (black line, $\lambda_{ex}$ = 297 nm). **(C)** Eu-CBP film (red line, $\lambda_{ex}$ = 260 nm) and CBP neat film (black line, $\lambda_{ex}$ = 267 nm). **(D)** Eu-T2T film (red line, $\lambda_{ex}$ = 260 nm) and T2T neat film (black line, $\lambda_{ex}$ = 267 nm).

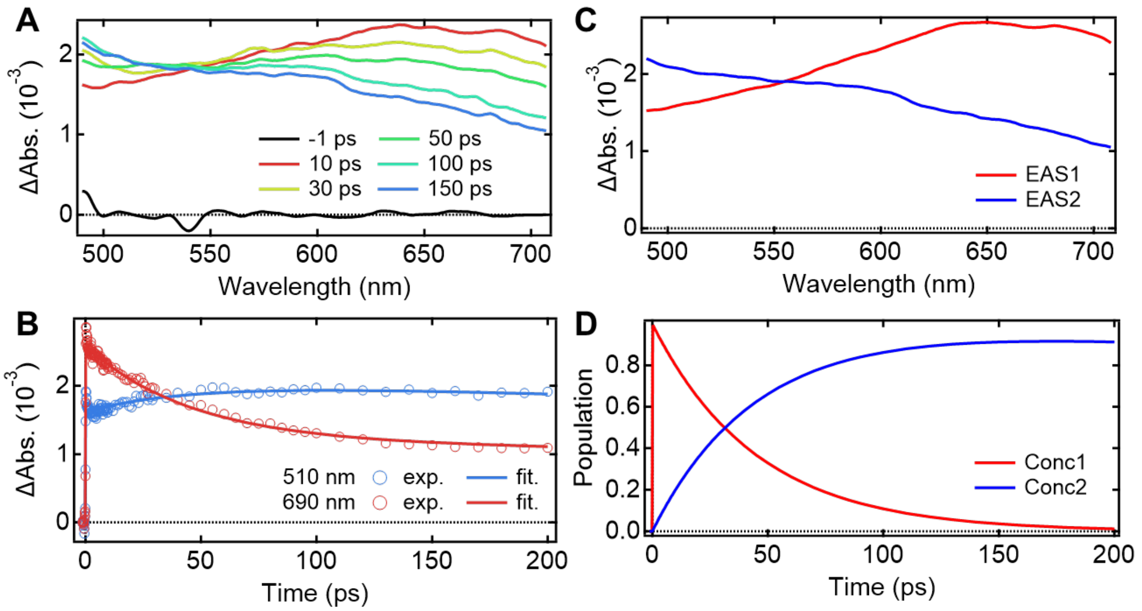


**Fig. S7. fs-TAS and their global analysis of the mT2T neat film. (A)** Temporal evolution of the fs-TAS spectra after photoexcitation at 267 nm. **(B)** Temporal profiles of the fs-TAS spectra at 510 nm (blue circles) and at 690 nm (red circles) and corresponding fitting curves resulted from the global analysis. **(C)** EAS and **(D)** Corresponding concentration kinetics obtained from the global analysis. The time constant of the transition from EAS1 to EAS2 was 44.9 ± 0.4 ps.

**Table S1. Optical properties of $Eu(hfa)_3(TPPO)_2$-doped films.** The decay rate constants of the $^5D_0$ state ($\boldsymbol{k_{\mathrm{obs}}^{\mathrm{Eu}}}$) are determined by the lifetime at 615 nm ($\lambda_{ex}$ = 320 nm). $\boldsymbol{I_{\mathrm{tot}}/I_{\mathrm{MD}}}$ is the ratio of the total area of the Eu(III) luminescence spectrum to the area of the $^5D_0 \rightarrow {}^7F_1$ transition band. Here, $\boldsymbol{n}$, $\boldsymbol{k_{\mathrm{r}}^{\mathrm{Eu}}}$, $\boldsymbol{k_{\mathrm{nr}}^{\mathrm{Eu}}}$, $\boldsymbol{\phi_{\mathrm{Eu}}}$, and $\boldsymbol{\eta_{\mathrm{sens}}}$ represent the refractive index of medium, the radiative rate constant of the Eu(III) ion, the non-radiative rate constant of the Eu(III) ion, the luminescence quantum yield of the Eu(III) ion, and the overall photosensitization efficiency, respectively.

| host | $k_{\mathrm{obs}}^{\mathrm{Eu}}$/s$^{-1}$ | $I_{\mathrm{tot}}/I_{\mathrm{MD}}$ | $n$ | $k_{\mathrm{r}}^{\mathrm{Eu}}$/s$^{-1}$ | $k_{\mathrm{nr}}^{\mathrm{Eu}}$/s$^{-1}$ | $\phi_{\mathrm{Eu}}$ | $\eta_{\mathrm{sens}}$ |
|---|---|---|---|---|---|---|---|
| PMMA | $1.2 \times 10^3$ | 21 | 1.5 | $1.0 \times 10^3$ | $1.8 \times 10^2$ | 0.85 | 0.71 |
| mT2T | $1.4 \times 10^3$ | 18 | 1.7 | $1.2 \times 10^3$ | $1.9 \times 10^2$ | 0.87 | 0.97 |
| SF3TRZ | $1.4 \times 10^3$ | 17 | 1.7 | $1.2 \times 10^3$ | $1.9 \times 10^2$ | 0.86 | 0.88 |
| mCBP | $1.4 \times 10^3$ | 15 | 1.7 | $1.1 \times 10^3$ | $2.9 \times 10^2$ | 0.79 | 0.42 |
| CBP | $1.4 \times 10^3$ | 15 | 1.7 | $1.1 \times 10^3$ | $3.4 \times 10^2$ | 0.76 | 0.38 |
| T2T | $1.4 \times 10^3$ | 17 | 1.8 | $1.3 \times 10^3$ | $1.1 \times 10^2$ | 0.92 | 0.17 |

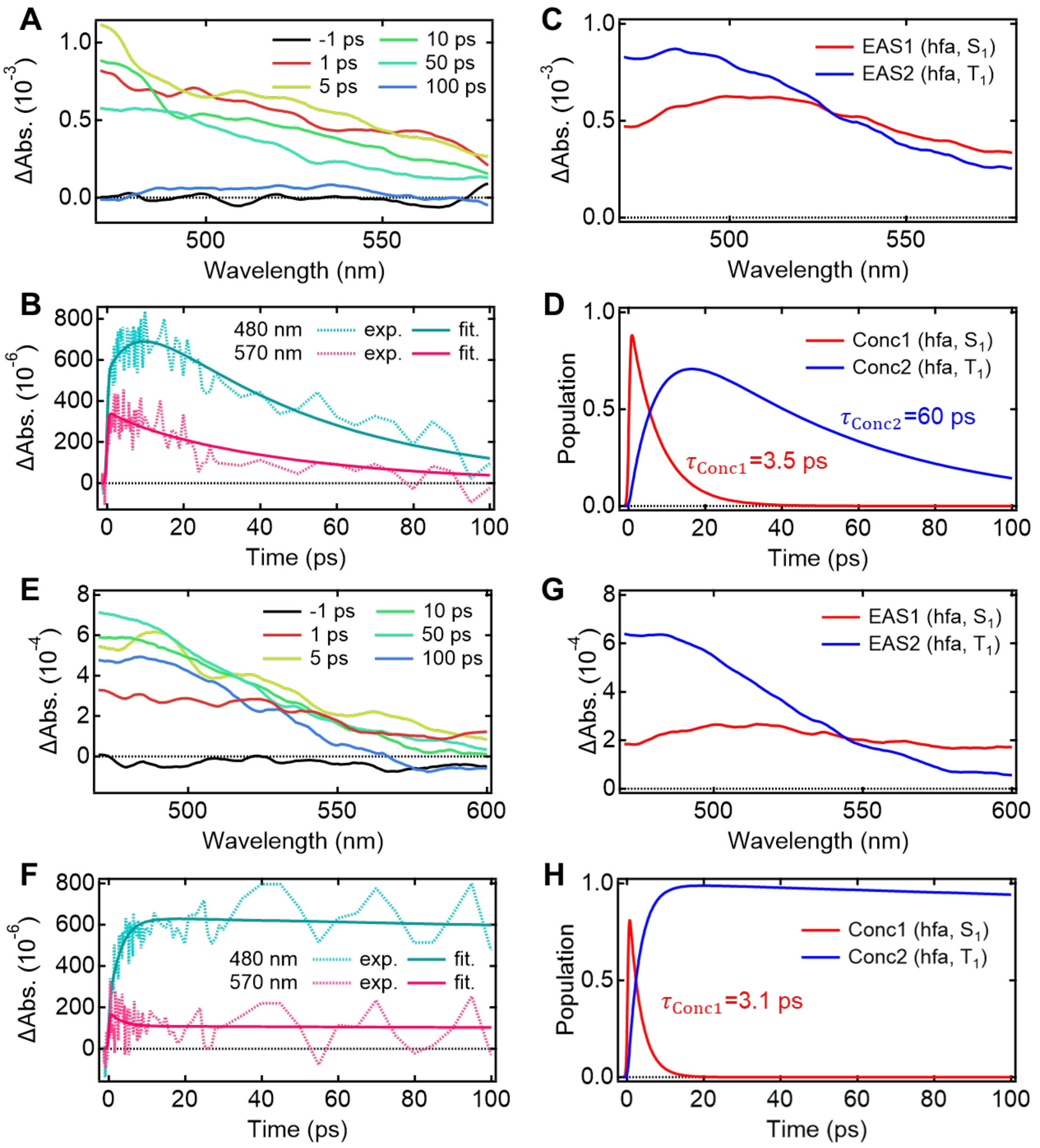


**Fig. S8. fs-TAS and their global analysis of neat films. (A-D)** Eu(hfa)$_3$(TPPO)$_2$ neat films **(E-H)** Gd(hfa)$_3$(TPPO)$_2$ neat film. **(A, E)** Temporal evolutions of the fs-TAS spectra after photoexcitation at 315 nm. **(B, F)** Temporal profiles of the fs-TAS spectra at 480 nm (green dotted line) and at 580 nm (pink dotted line), and the corresponding fitting curves resulted from the global analysis. **(C, G)** EAS and **(D, H).** Corresponding concentration kinetics were obtained from the global analysis based on sequential models (*4*).

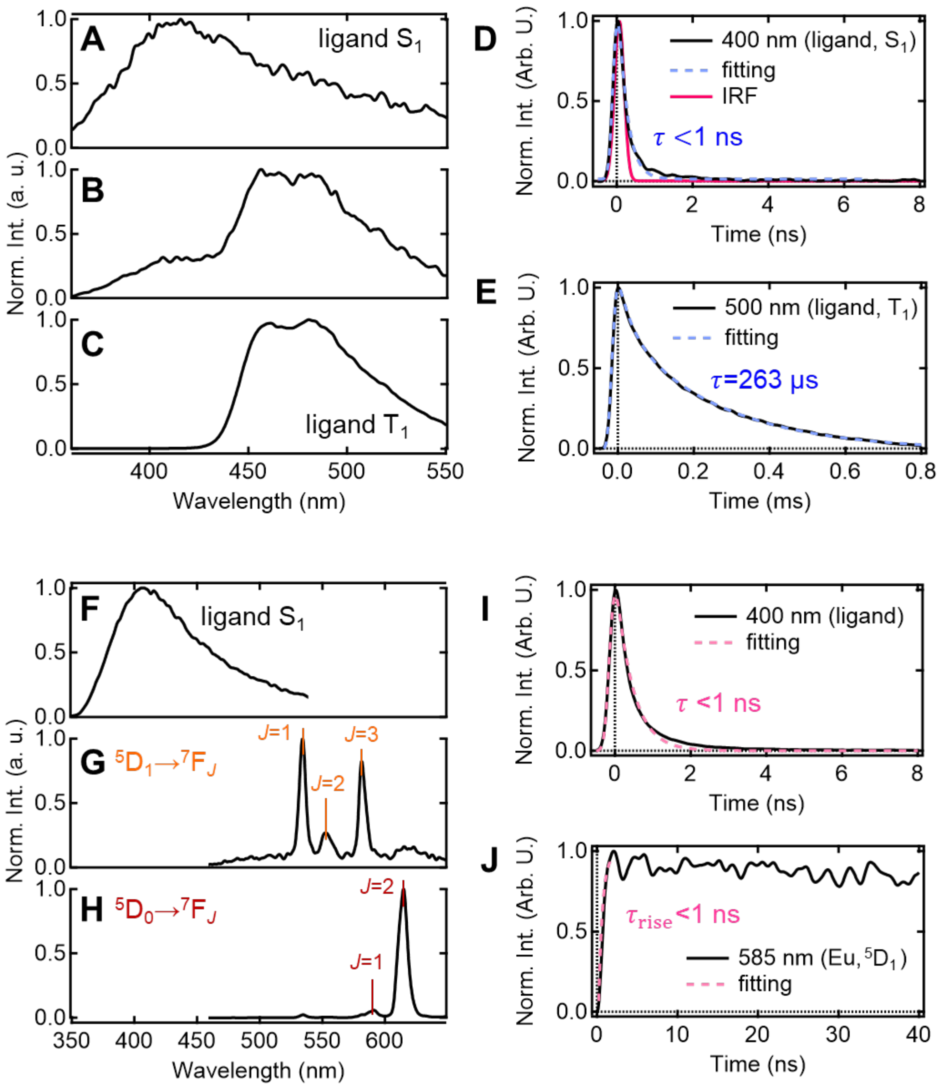


**Fig. S9. TR-PL and their analysis of neat films. (A-E)** TR-PL data of Gd(hfa)$_3$(TPPO)$_2$ neat film. Integrated spectra at **(A)** 0–1 ns, **(B)** 80–100 ns, and **(C)** 10–30 µs. **(D)** Temporal profile at 400 nm (black solid line), which is assigned to the fluorescence from the hfa ligands, and fitting curve assuming a single exponential decay (sky blue dashed line), and instrumental response function (IRF). **(E)** Temporal profile at 500 nm (black solid line), which is assigned to the phosphorescence from the hfa ligands, and a fitting curve assuming a single exponential decay (sky blue dashed line). **(F-J)** TR-PL data of the Eu(hfa)$_3$(TPPO)$_2$ neat film. Integrated spectra at **(F)** 0–1 ns, **(G)** 10–20 ns, and **(H)** 6–8 µs. **(I)** Temporal profile at 400 nm, which is assigned to the fluorescence from hfa ligands, and a fitting curve assuming a double exponential decay (pink dashed line). **(J)** Temporal profile at 585 nm (black solid line), which is assigned to the emission from $^5D_1$ of the Eu(III) ion, and fitting curve assuming a single exponential rise (pink dashed line).

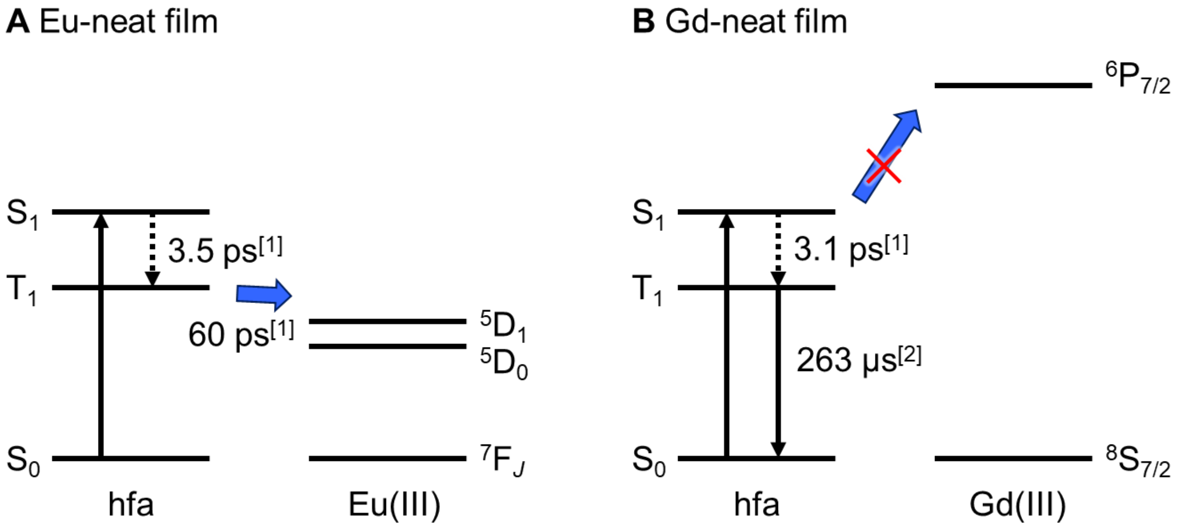


**Fig. S10. Schematic diagram of intra-molecular energy transfer in neat films. (A)** Eu(hfa)$_3$(TPPO)$_2$-doped film. **(B)** Gd(hfa)$_3$(TPPO)$_2$-doped neat film. The rate constants were determined by fs-TAS[1] (Fig. S8) and TR-PL[2] measurements (Fig. S9).

**Table S2. Lifetimes of the $T_1$ state of the ligands and intra-molecular energy transfer efficiency.** The rate constants ($\boldsymbol{k_{\mathrm{L}}^{\mathrm{T_1}}}$) were determined by the lifetimes of the $T_1$ of the hfa ligands in the $Eu(hfa)_3(TPPO)_2$ and $Gd(hfa)_3(TPPO)_2$ neat films (Fig. S10) after photoexcitation of hfa (excitation wavelength: $\lambda_{ex} = 315$ nm). The efficiencies of the intra-molecular energy transfer ($\boldsymbol{\kappa_{\mathrm{ET}}}$) were calculated by Eq. (S5).

| guest | $k_{\mathrm{L}}^{\mathrm{T_1}}$/s$^{-1}$ | $\kappa_{\mathrm{ET}}$ |
|---|---|---|
| $Eu(hfa)_3(TPPO)_2$ | $1.7 \times 10^{10}$ | >0.99 |
| $Gd(hfa)_3(TPPO)_2$ | $3.8 \times 10^{3}$ | - |

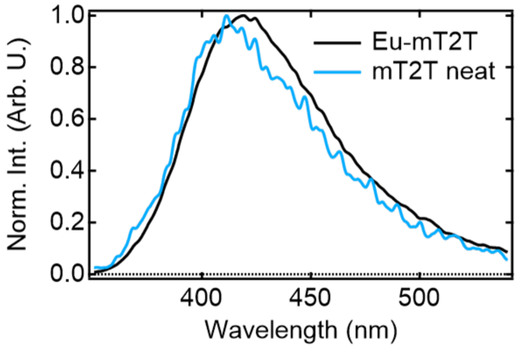


**Fig. S11. Comparison of TR-PL spectra between the doped and neat films.** Integrated TR-PL spectra of the Eu-mT2T film at 0–10 ns (black line) and the mT2T neat film at 0–10 ns (sky blue line). The excitation wavelength was 267 nm. The spectrum of the Eu-mT2T film is assigned to the fluorescence from mT2T.

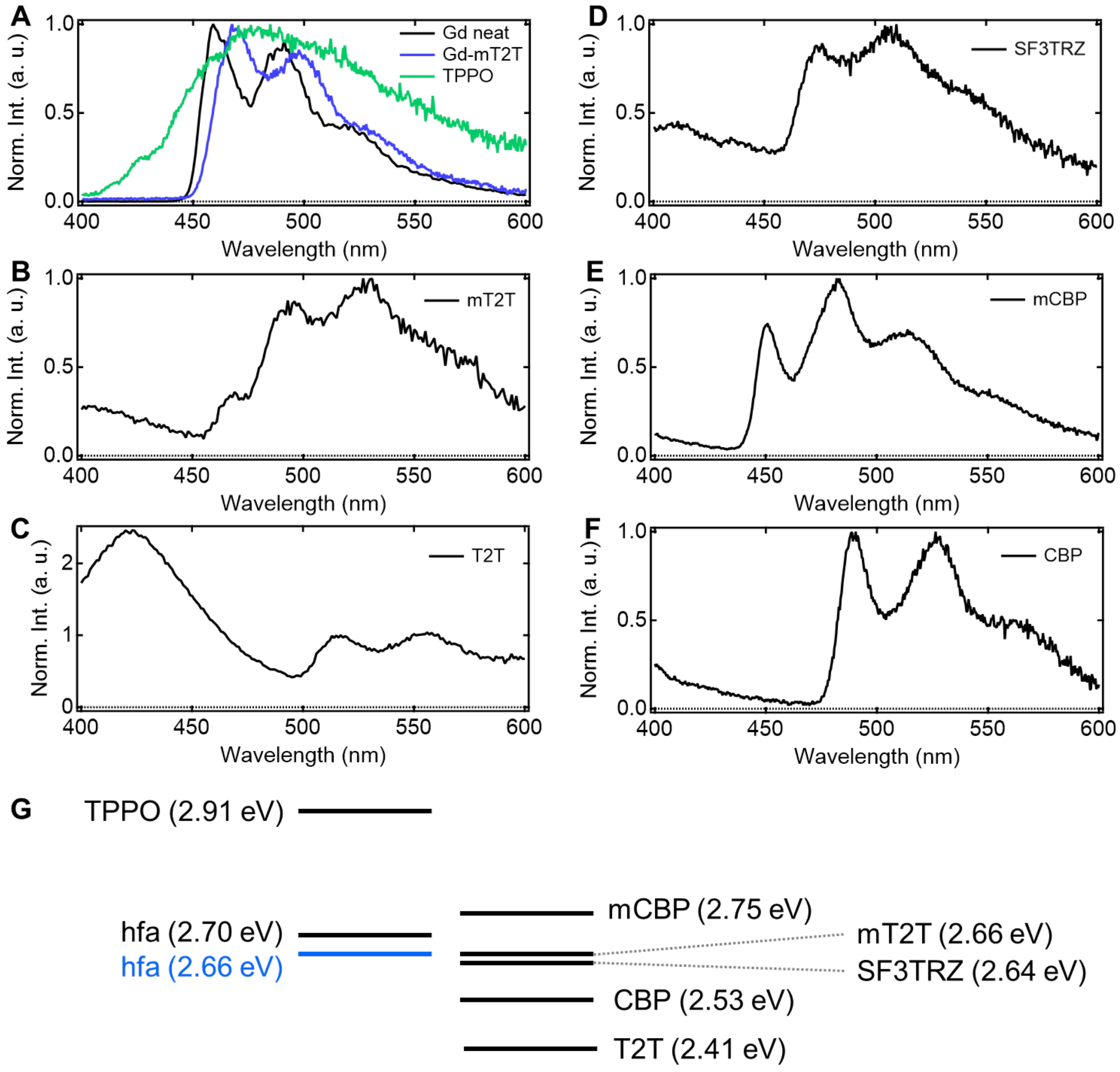


**Fig. S12. Phosphorescence spectra of host molecules and ligands measured at 77 K. (A)** $Gd(hfa)_3(TPPO)_2$ neat film (black line), $Gd(hfa)_3(TPPO)_2$-doped mT2T film (blue line), and TPPO neat film (green line). **(B)** mT2T, **(C)** pT2T, **(D)** SF3TRZ, **(E)** mCBP, and **(F)** pCBP neat films. **(G)** Schematic energy diagram of the $T_1$ excited states estimated from the phosphorescence spectra. Note that the energy level of hfa ligands in the $Gd(hfa)_3(TPPO)_2$ neat film (black line) differs from that in the $Gd(hfa)_3(TPPO)_2$-doped mT2T film (blue line).

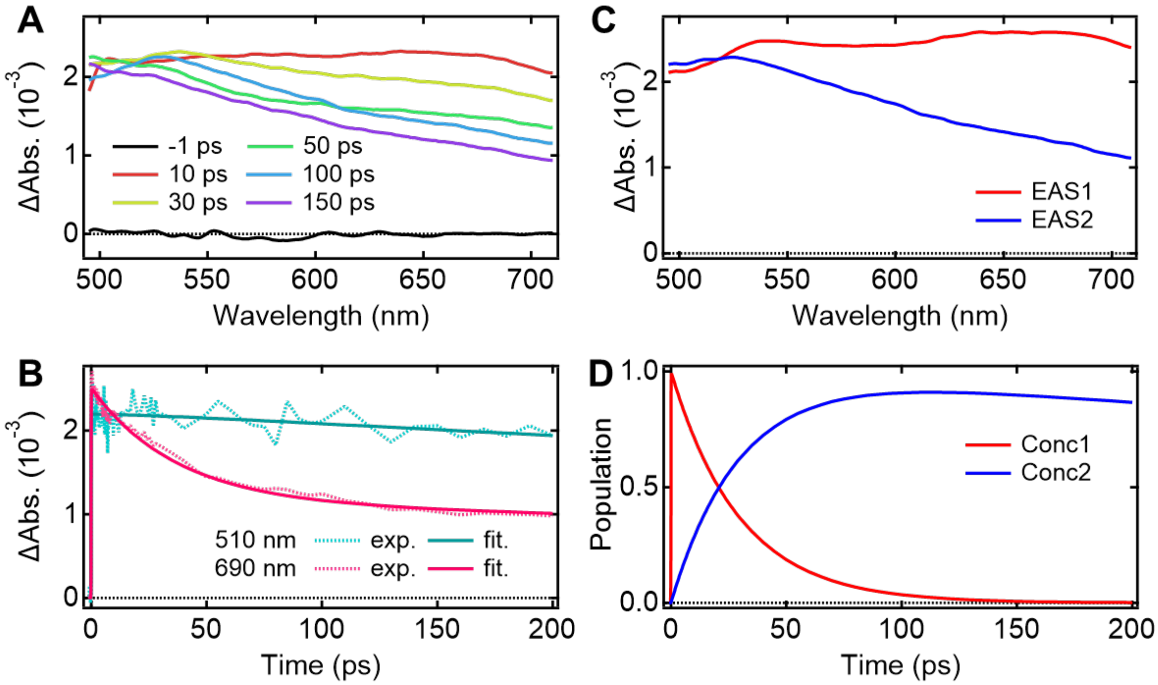


**Fig. S13. fs-TAS and their global analysis of the Gd-mT2T film. (A)** Temporal evolution of the fs-TAS spectra after photoexcitation at 267 nm. **(B)** Temporal profiles at 510 nm (green dotted line) and at 690 nm (pink dotted line). The corresponding fitting curves resulted from the global analysis. **(C)** EAS and **(D)** corresponding concentration kinetics were obtained from the global analysis based on sequential models (*4*). The transition timescale from EAS1 to EAS2 was 36.3 ± 0.3 ps.

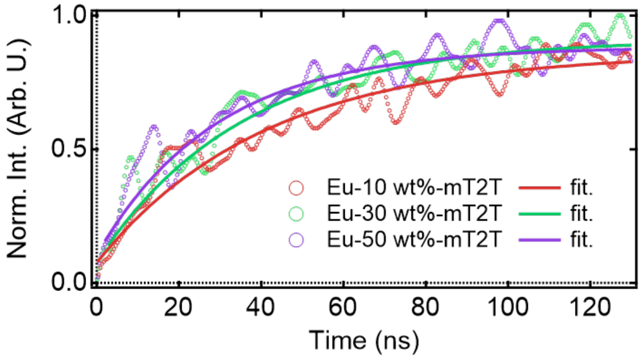


**Fig. S14. Comparison of temporal profiles of emission from the Eu(III) ion in a doped film with different doping ratios.** Temporal profiles of the TR-PL spectra at 585 nm, which is assigned to the emission of $^5D_1$ of the Eu(III) ion in Eu(hfa)$_3$(TPPO)$_2$ 10 wt%-doped Eu-mT2T (Eu-10 wt%-mT2T, red circle) film, 30 wt%-doped Eu-mT2T (Eu-30 wt%-mT2T, green circle) film, and 50 wt%-doped Eu-mT2T (Eu-50 wt%-mT2T, purple circle) film. The corresponding solid curves are fitting curves assuming a single exponential rise. The obtained rise time constant of the Eu-10 wt%-mT2T film is 41.0 ± 0.8 ns, the rise time constant of the Eu-30 wt%-mT2T film is 35.5 ± 1.2 ns, and the rise time constant of the Eu-50 wt%-mT2T film is 29.1 ± 1.0 ns.

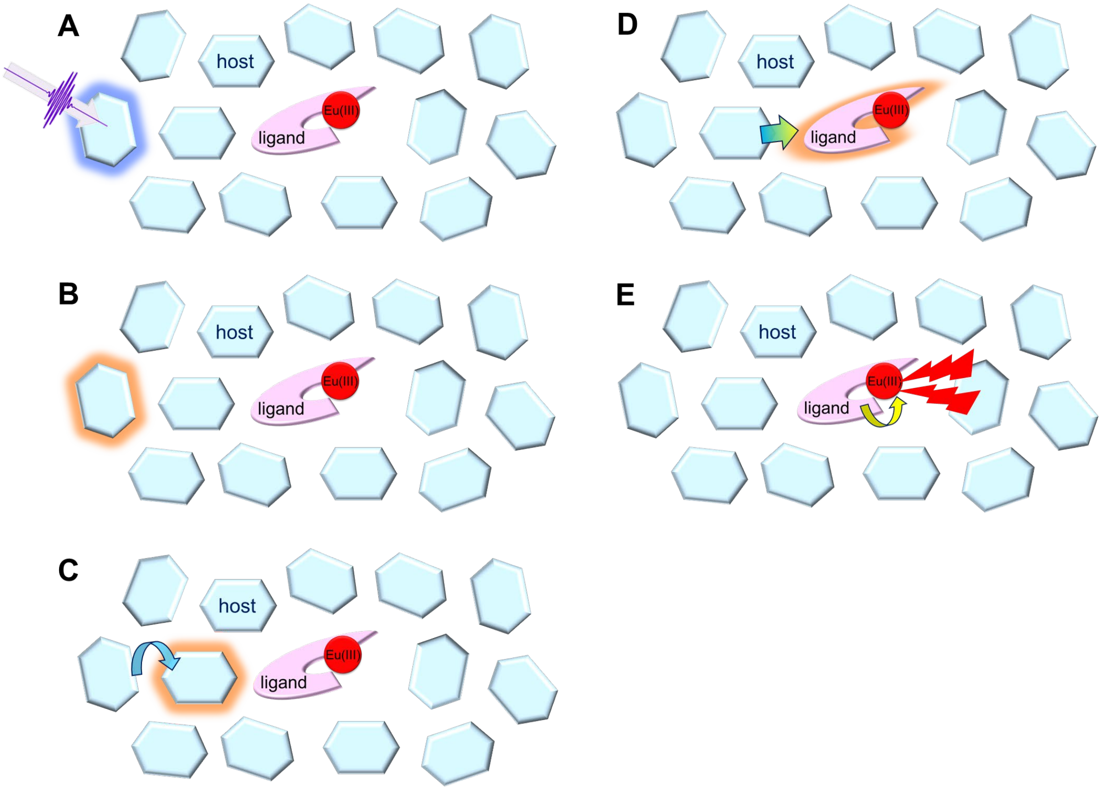


**Fig. S15. Overview of energy transfer processes in the Eu-mT2T film. (A)** Photoexcitation of a mT2T host molecule. **(B)** ISC occurs in the mT2T host molecule. **(C)** Excitation diffusion between mT2T occurs in ~40 ns. **(D)** Intermolecular energy transfer occurs from the nearest mT2T generated from the excitation diffusion to a hfa ligand of a Eu(III) complex. **(E)** Intramolecular energy transfer occurs from the ligand to the Eu(III) ion.

**Table S3. Environmental dependency of PLQY of Eu(III)-complex-doped films.** PLQYs were measured under ambient atmosphere or under the argon (Ar) atmosphere.

| guest | host | Eu conc. /wt% | environment | $\boldsymbol{\phi}_{\mathbf{tot}}$ | $\boldsymbol{\lambda}_{\mathbf{ex}}$ /nm |
|---|---|---|---|---|---|
| $Eu(hfa)_3(TPPO)_2$ | PMMA | 10 | ambient | 0.60 | 315 |
| | | | Ar | 0.61 | |
| | mT2T | 10 | ambient | 0.79 | 267 |
| | | | Ar | 0.84 | |
| | | 30 | ambient | 0.78 | 267 |
| | | | Ar | 0.83 | |
| | | 50 | ambient | 0.77 | 267 |
| | | | Ar | 0.78 | |

**Table S4. Film thicknesses of the host-guest films.** The concentration of $Eu(hfa)_3(TPPO)_2$ in the host-guest films is 10 wt%.

| guest | host | Thickness (nm) |
|---|---|---|
| $Eu(hfa)_3(TPPO)_2$ | - | 83.0 |
| | PMMA | 166 |
| | mT2T | 118 |
| | SF3TRZ | 117 |
| | mCBP | 123 |
| | pCBP | 124 |
| | pT2T | 97.5 |

## References


1. M. H. V. Werts, R. T. F. Jukes, J. W. Verhoeven, *Phys. Chem. Chem. Phys.*, 2002, **4**, 1542-1548.

2. G. D. R. Napier, J. D. Neilson, T. M. Shepherd, *Chem. Phys. Lett.*, 1975, **31**, 328–330.

3. Y. C. Miranda, L. L. A. L. Pereira, J. H. P. Barbosa, H. F. Brito, M. C. F. C. Felinto, O. L. Malta, W. M. Faustino, E. E. S. Teotonio, *Eur. J. Inorg. Chem.*, 2015, **18**, 3019–3027.

4. J. J. Snellenburg, S. P. Laptenok, R. Seger, K. M. Mullen, I. H. M. van Stokkum, *Journal of Statistical Software*, 2012, **49** *(3)*, 1-22.
